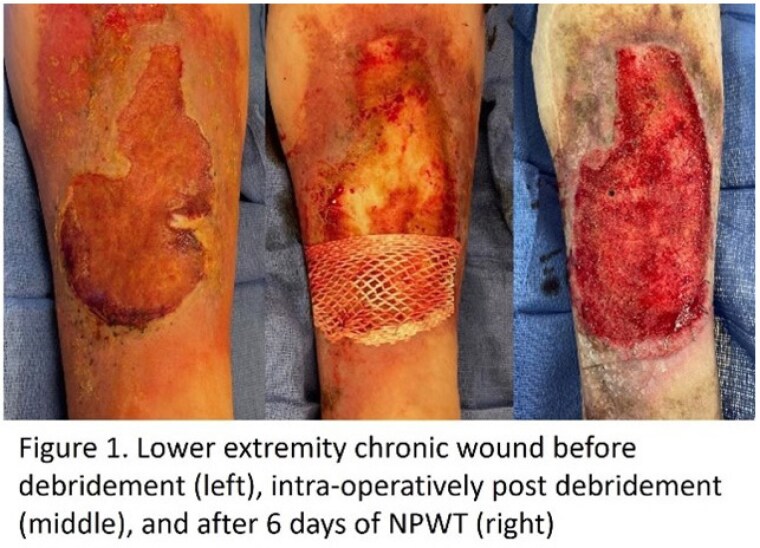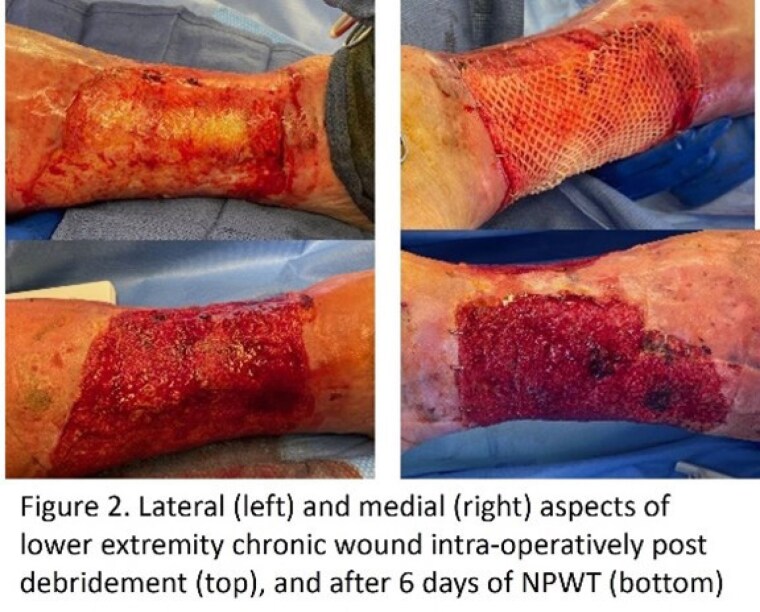# 534 Does Intact Fish Xenograft Assist in Wound Bed Preparation: A Retrospective Evaluation

**DOI:** 10.1093/jbcr/iraf019.163

**Published:** 2025-04-01

**Authors:** Matthew Supple, Paul Comish, Kiran Dyamenahalli, Sean Hickey, John Schulz, Jeremy Goverman

**Affiliations:** Massachusetts General Hospital; Massachusetts General Hospital; Massachusetts General Hospital; Massachusetts General Hospital; Massachusetts General Hospital; Massachusetts General Hospital

## Abstract

**Introduction:**

Excision and autografting are frequently staged to allow time for an increase in wound bed vascularity via formation of granulation tissue. Negative pressure wound therapy (NPWT) and skin substitutes are frequently used to regenerate tissue, stimulate a wound bed, and hasten granulation formation. Intact fish skin (FS) is a xenograft, biologic, skin substitute reported to regenerate tissue, decrease length of stay, minimize prolonged hospitalization, and improve resource allocation. We hypothesized that NPWT is equally effective in creating a vascularized wound bed with or without the use of an underlying FS.

**Methods:**

We retrospectively reviewed our demographic reports for patients that underwent excisional debridement followed by placement of FS and NPWT for either burn or chronic wounds. Photos were reviewed to find patients for whom part of the wound bed was not covered completely with xenograft, leaving a portion of the wound bed open for direct contact with NPWT foam. Photos before and after NPWT were cropped, sized, categorized as with or without FS; and burn care providers were asked to identify which wound bed appeared more appropriate for grafting, or if equal in appearance.

**Results:**

Six paired wound bed photos (2 burn, 4 chronic wound) were reviewed by 10 practitioners. Overall, there was no preference with respect to FS + NPWT vs NPWT alone. 24% of overall responses reported wound beds as equal in appearance.

**Conclusions:**

It is possible that the use of FS with NPWT for wound bed preparation may not offer advantages over the use of NPWT alone. In this small, uncontrolled, cohort, the prepared wound beds were deemed to appear macroscopically similar. Prospective randomized controlled trials comparing NPWT with and without the use of various skin substitutes are needed, as these products increase overall costs.

**Applicability of Research to Practice:**

This study raises the important question of how skin substitutes assist in wound bed preparation and hopes to challenge clinicians to conduct further studies on this common technique.

**Funding for the Study:**

N/A